# P-863. Incidence and Cumulative Risk Factors for Prolonged QTc Interval in Cirrhotic Patients Receiving Fluoroquinolone Prophylaxis

**DOI:** 10.1093/ofid/ofaf695.1071

**Published:** 2026-01-11

**Authors:** Thomas Pustorino, Kelsey McManus, Nicholas Feola, Abhay Dhand

**Affiliations:** Westchester Medical Center, Valhalla, New York; Westchester Medical Center, Valhalla, New York; Westchester Medical Center, Valhalla, New York; Westchester Medical Center, Valhalla, New York

## Abstract

**Background:**

Fluroquinolone (FQ) antibiotics are currently the acceptable therapy for spontaneous bacterial peritonitis (SBP) prophylaxis in selected patients with cirrhosis. Based on additional comorbidities and fluid/electrolyte imbalance, these patients may have multiple other risk factors for prolonged corrected QT interval (QTc) beyond a low dose FQ therapy.

**Figure:**
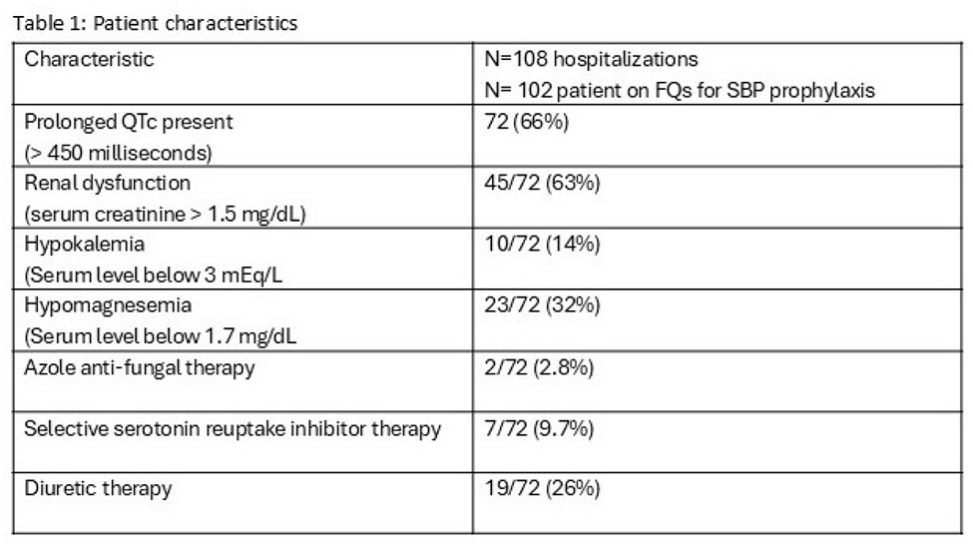

**Figure:**
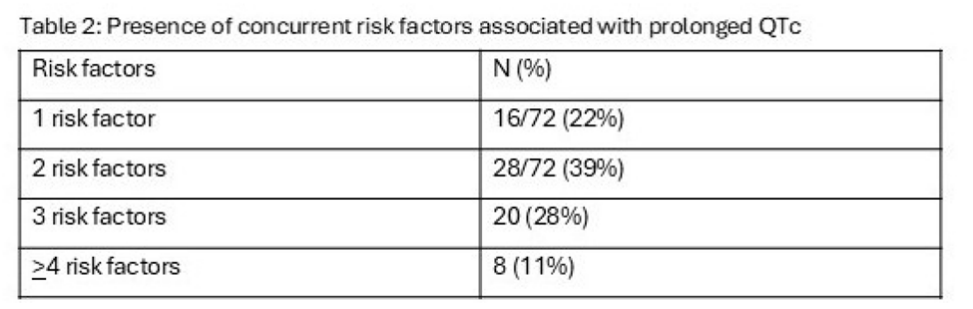

**Methods:**

Consecutive adult cirrhotic patients hospitalized from June 2022 to June 2024 who were on FQ therapy for SBP prophylaxis for at least 72 hours with an available electrocardiogram (ECG) were included in the study. Data was collected for QTc interval, patient characteristics, along with the presence of additional concurrent risk factors known to be associated with a prolonged QTc including kidney dysfunction, serum potassium and magnesium levels and other medications (anti-psychotics, azoles and/or diuretics). Outcomes measures were incidence of prolonged QTc, number of cumulative risk factors associated with prolonged QTc and any documented ventricular arrythmia.

**Results:**

During the study period,102 (36% female) patients with 108 unique hospitalizations who met the study criteria were identified. Majority patients were on low dose ciprofloxacin (250 mg once a day) for an average of 8.3 days (range 3-30) during their hospitalization. Data is summarized in tables 1 & 2. None of the patients had a documented ventricular arrythmia event during their hospitalization.

**Conclusion:**

Patients with cirrhosis are at risk of poly-pharmacy and multiple organ dysfunction resulting in multiple risk factors for prolonged QTc. Among 108 consecutive hospitalizations among patients who were on low dose FQ therapy of SBP prophylaxis, 66% were found to have a prolonged QTc on routine EKG documented without any evidence of documented ventricular arrythmia event during their hospitalization. Active identification and treatment of potentially modifiable risk factors associated with prolonged QTC may help prevent any cardiac rhythm complications in patients with cirrhosis.

**Disclosures:**

Abhay Dhand, MD, Eurofins Viracor: Advisor/Consultant|Eurofins Viracor: Honoraria|Merck: Advisor/Consultant|Merck: Honoraria

